# Resection arthroplasty versus dual mobility prosthesis in the treatment of trapeziometacarpal joint osteoarthritis: A 3 year non-randomized prospective study

**DOI:** 10.1016/j.jor.2024.06.005

**Published:** 2024-06-10

**Authors:** Florian Falkner, Arman Mahmut Tümkaya, Benjamin Thomas, Arne Böcker, Martin Aman, Berthold Bickert, Leila Harhaus, Benjamin Panzram

**Affiliations:** a Department of Hand and Plastic Surgery, Peripheral Nerve Surgery and Rehabilitation, BG Trauma Center Ludwigshafen, Ludwig-Guttmann-Strasse 13, 67071, Ludwigshafen, Germany; b Department of Hand and Plastic Surgery, Heidelberg University, Im Neuenheimer Feld 420, 69120, Heidelberg, Germany; c Department of Orthopaedics, University of Heidelberg, Schlierbacher Landstraße 200a, 69118, Heidelberg, Germany

**Keywords:** Trapeziometacarpal osteoarthritis, TMC joint, Arthroplasty, Prosthesis, Dual mobility

## Abstract

**Purpose:**

Resection arthroplasty (RA) is still the most common surgical intervention for the treatment of symptomatic trapeziometacarpal (TMC) joint osteoarthritis. The implantation of a dual mobility prosthesis may represent a joint function preserving alternative. The aim of the presented study is to prospectively compare the outcomes of RA with dual mobility prosthesis.

**Methods:**

In this 2-center non-randomized prospective study, we compared results of RA (n = 22) with implantation of a dual mobility prosthesis (n = 49) (Touch®) at a minimum of 3-year follow-up. The patients underwent preoperative assessments and postoperative follow-up at 6 weeks, 3, 6, 12, 24, and 36 months. Comparisons were conducted, covering pain assessment via the visual analogue scale (VAS), thumb range of motion (ROM), pinch and grip strength, as well as functional scores and radiological examinations.

**Results:**

The time intervals from surgery until absence of pain on the VAS (3 months: 3 vs 1, p = 0.0001), recovery of ROM in radial (3 months: 33° vs 42°, p = 0.0001), and palmar abduction (3 months: 33° vs 48°, p = 0.0001), were significantly longer for the RA group compared with the prosthesis group. At 3-year follow-up there was no significant difference in absence of pain, ROM and grip strength between both groups. Key pinch strength was significantly weaker in the RA group compared to prosthesis group at 3 months (2.6 kg vs 4.6 kg, p = 0.001), to 3-year follow-up (3.1 kg vs 5.7 kg, p = 0.0001). The final mean DASH (15.5 vs 13.2, p = 0.01) and MHQ scores (78 vs 82, p = 0.01) were significantly better in the prosthesis group.

**Conclusion:**

Both techniques show high patient satisfaction in mid-term follow-up. Dual mobility TMC joint arthroplasty seems to be associated with a superior pinch strength and shorter time of recovery as compared to patients after RA.

## 1 Introduction

Osteoarthritis of the trapeziometacarpal (TMC) joint is a prevalent condition, especially among postmenopausal women.^1^ When symptoms are present and patients do not respond to conservative treatment, surgical procedures are considered. Resection arthroplasty (RA) has been the standard procedure for a long time.^2^^,^^3^ However, this procedure is highly invasive as it involves the removal of the trapezium. RA can have a negative impact on the thumb's biomechanical stability due to reduction of thumb length. In addition, the period of recovery and the return to pain-free movements can be relatively long, taking several weeks or even months. Furthermore, proximalisation of the first metacarpal can result in impingement on the scaphoid, leading to a subsequent decrease of grip strength and pinch strength.^4^ Finally, in the case of RA related complications, there are still no satisfactory options for management.

An alternative that preserves the trapezium is TMC joint arthroplasty using a dual mobility prosthesis. This procedure replaces the TMC joint with the aim of maintaining normal thumb length to enhance grip strength and stability.^5^^,^^6^ In addition, it prevents the first metacarpal bone from subsidence. To compare the mid-term results of the two techniques, we conducted a prospective 2-center study comparing functional and radiographic outcome after total TMC joint arthroplasty with those after conventional RA.

## 2 Material and methods

### 2.1 Study design and inclusion criteria

Following IRB approval, a non-randomized prospective study was carried out in two hospitals. From July 2018 to September 2020, every patient who fulfilled the indication for operative treatment of TMC osteoarthritis and the requirements for implantation of a dual mobility prosthesis was informed about the proven concept of traditional trapezium resection arthroplasty and about the innovative concept of implantation of a dual mobility prosthesis, the long-term results (>10 years) of the latter still being unknown. Thus, the inclusion criteria were the following: (1) Primary symptomatic degenerative TMC joint osteoarthritis with unsuccessful conservative therapy. (2) Primary osteoarthritis (Eaton and Littler stage II or III). (3) Trapezium height of at least 9 mm. (4) Patient profile indicating moderate physical hand loading. (5) No previous surgery at the TMC joint. None of the patients had received intraarticular cortisone injections previously. Exclusion criteria included (1) stage I osteoarthritis, (2) concomitant scapho-trapezio-trapezoidal joint osteoarthritis (Eaton Glickel stage IV osteoarthritis), and (3) patient's denial to participate in the study.

After being provided with detailed information about RA and dual mobility arthroplasty as surgical options all included patients were given the freedom to choose their preferred surgical treatment. Out of a total of 69 patients and 71 thumbs fulfilling the inclusion criteria, 47 patients (49 thumbs) decided for prosthesis implantation (prosthesis group) whereas 22 patients wished a resection arthroplasty (RA group). RA was performed in alle patients without ligament reconstruction and tendon interposition. Two patients in the Prosthesis group had bilateral prostheses one after the other. The prosthesis used in all thumbs was the TOUCH® dual mobility trapeziometacarpal prosthesis, KeriMedical, Les Acacias, Switzerland. Possible surgical complications were taken from the operation records.

### 2.2 Postoperative care

After RA the thumb was immobilized in a brace, for six weeks. Following this period, active thumb mobilization was initiated under the supervision of a physical therapist. Thumbs undergoing TMC joint arthroplasty were immobilized in a brace for one week. Subsequently, active thumb mobilization was continued with assistance from a physical therapist. Full weight bearing was allowed after six weeks in both groups. During a minimum postoperative follow-up of 3 years, functional and radiologic outcomes of all patients were recorded.

### 2.3 Clinical and radiological follow-up examinations

The patients included in the study underwent preoperative assessments and postoperative follow-up at 6 weeks, 3, 6, 12, 24, and 36 months. Pain was recorded using a visual analogue scale (VAS) that ranged from 0 (indicating no pain) to 10 (representing the worst imaginable pain) during specific activities like "bottle cap opening" or "door opening with a key". Joint range of motion (ROM) of the TMC joint in radial and palmar abduction was measured using standard goniometers. Opposition of the thumb was evaluated using the Kapandji score.^7^ Grip strength and key pinch strength were assessed using the Jamar dynamometer and pinch meter, respectively (Jamar Technologies Inc. in Clifton, NJ, USA).

Overall hand and upper extremity function was assessed using the Quick Disabilities of the Arm, Shoulder, and Hand (QuickDASH) questionnaire, and the Michigan Hand Questionnaire (MHQ).^8^^,^^9^ Quality of life was assessed with the 36-Item Short Form Survey (SF-36).^10^

**Fig. 1:**
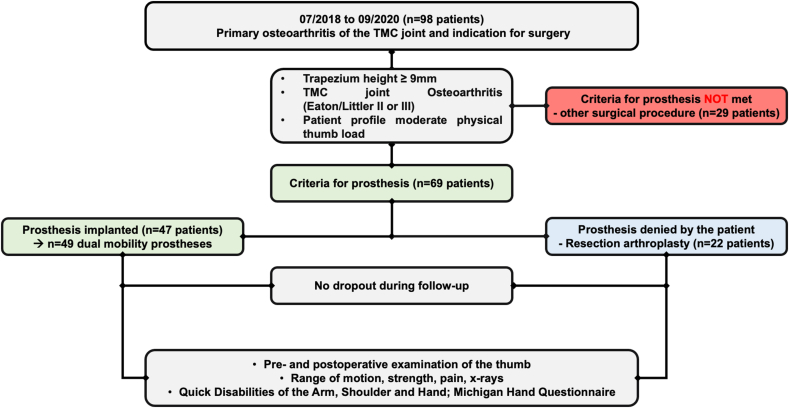
Flowchart of the study design and patient inclusion.

Entire thumb X-ray were captured in both posterior-anterior and lateral views before surgery, immediately post-operation, and during each subsequent follow-up appointment. Radiographs were investigated for radiolucent areas in the metacarpal or trapezium bones, and for dislocation or loosening of the implant, or for impingement of the first metacarpal bone and for remnants of the trapezium, respectively. Postoperative thumb length restoration was examined by measuring the distance from the distal pole of the scaphoid to the thumb tip on posterior-anterior views in both groups using the DeepUnity X-ray viewer (Dedalus Healthcare Group AG, Bonn, Germany). The study design is illustrated in Fig. 1.

### 2.4 Statistical analysis

A result was considered significant if the p value was less than 0.05. Continuous variables between the two groups were compared using an analysis of variance (two-way repeated measures ANOVA) for interval-scaled values at different follow-up time points, together with an independent *t*-test. Results were reported as means with standard deviations. To control surgical confounders identified (dominant hand, operative time, gender, stage of arthrosis, age, preoperative values) a multiple linear regression was calculated.

## 3 Results

### 3.1 Comparison of range and motion

**Fig. 2:**
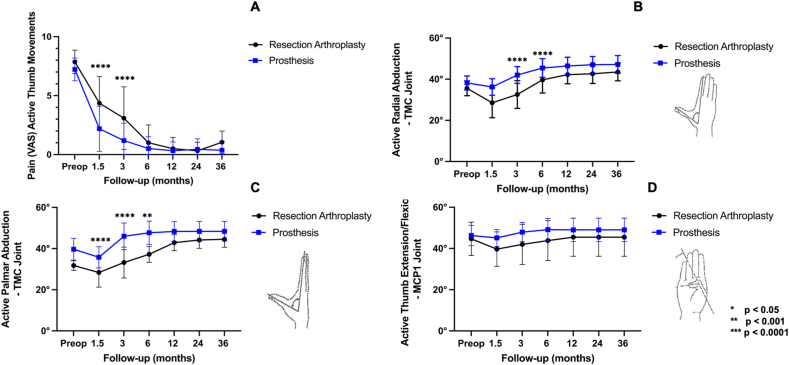
Graphs comparing means (error bars: SD) of resection arthroplasty and dual mobility prosthesis. (A) Visual Analogue Scale (VAS) for pain experienced during active thumb movements, (B) active radial abduction and (C) active radial abduction at the trapeziometacarpal (TMC), (D) range of motion (ROM) for active extension and flexion of the metacarpophalangeal 1 joint (MCP1) of the thumb.

69 patients (RA: n = 22; prosthesis: n = 49, 2 bilateral) were included in the study completing a 3-year follow-up. No patients dropped out. Both groups did not differ regarding their demography, comorbidities, and stage of osteoarthritis as shown in Table 1. The time from surgery until pain resolved was significantly shorter in the prosthesis group compared with the RA group. (1.5 months (mean difference on the VAS: 2.2; 95 % CI: 1.1 to 3.3; p = 0.19), and 3 months (mean difference on the VAS: 1.9; 95 % CI: 0.8 to 3.1); p < 0.0001). At 3-year follow-up pain reduction showed comparable results in both groups (mean difference on the VAS: 0.7; 95 % CI: −0.5 to 1.8; p = 0.7). In the first 6 months postoperatively radial and palmar abduction showed a difference but comparable results at 3-year follow-up (radial abduction - mean difference: 3.6°; 95 % CI: 0.3 to 7.6; p = 0.1; palmar abduction - mean difference 3.9°; 95 % CI: 0.7 to 8.7; p = 0.2) (Fig. 2). Thumb opposition improved and was comparable during the 3-year follow-up period in both groups (3 year mean difference: 0.1; 95 % CI: −1.1 to 0.9; p = 0.9).

**Table 1:** Patient characteristics and comorbidities of patients with RA and dual mobility Touch® prosthesis.

Patient Details	Total	Resection Arthroplasty	Prosthesis
Patients	69	22	47 (n = 49 prostheses)
Age (years)	57 (41–76)	56 (41–73)	58 (42–76)
Gender (women/men)	38/14	19/3	35/12
Operative time (minutes)	75 (49–140)	73 (55–121)	76 (49–140)
Dominant hand	35 (51 %)	13 (59 %)	22 (47 %)
Eaton and Littler Stage	II: 13 (18 %) III: 58 (82 %)	II: 5 (23 %) III: 17 (77 %)	II: 8 (16 %) III: 41 84 %)
Working/retired	58/11	19/3	39/8
Comorbidities:			
Arterial hypertension	8 (12 %)	2 (9 %)	6 (13 %)
Chronic obstructive pulmonary disease	5 (7 %)	2 (9 %)	3 (6 %)
Active smoker at time of surgery	5 (7 %)	2 9 %)	3 (6 %)
Coronary Heart Disease	2 (3 %)	0	2 (4 %)

### 3.2 Comparison of grip and pinch strength

**Fig. 3:**
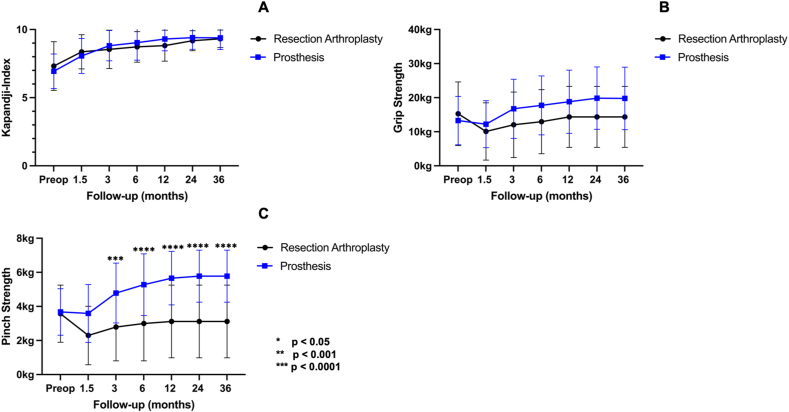
Graphs comparing means (error bars: SD) of resection arthroplasty and dual mobility prosthesis during follow-up intervals. (A) Kapandji index, (B) grip- and (C) pinch strength.

Mean grip strength values were comparable for both groups and showed no significant difference (3 year mean difference: 5.4 kg; 95 % CI: 2.1 to 12.9; p = 0.5). Mean pinch grip was significantly stronger in the prosthesis group at all timepoints of examination (3 year mean difference 2.7 kg; 95 % CI: 1.2 to 4.2; p < 0.0001) (Fig. 3). A multiple linear regression analysis revealed that the operative method (RA vs P) was the only variable significantly correlating with the postoperative improvement of pinch strength at 3-year follow-up (β = 0.23; p < 0.0001). The mean values including standard deviation of pain, ROM, and strength for both groups during follow-ups are summarized in Table S1.

### 3.3 Comparison of functional scores

**Fig. 4:**
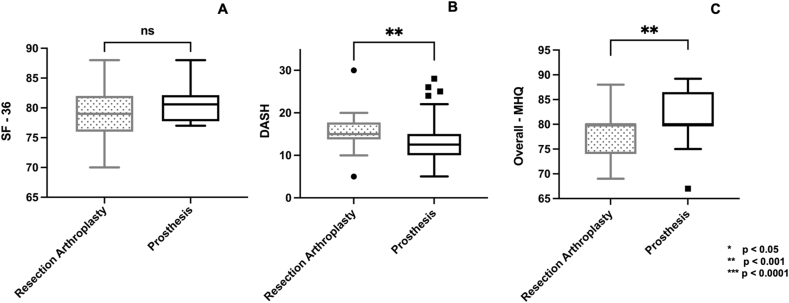
Boxplot graphs (minimum, 25th percentile, median, 75th percentile, maximum) illustrate the comparison of functional scores between resection arthroplasty and dual mobility prosthesis 3 years after surgery. (A) The 36-Item Short Form Survey (SF-36) for quality of life, (B) QuickDASH and (C) the Michigan Hand Questionnaire (MHQ).

The 3 years mean DASH score and mean MHQ score were significantly better in the prosthesis group ((DASH: RA - 15.4 (range 6–30) vs prosthesis - 13.2 (range 6–28); p = 0.01; MHQ: RA - 78 (range 69–88) vs prosthesis - 82 (range 67–89); p = 0.01)). SF-36 showed no difference between both groups (Fig. 4). Further, the return-to-work duration was significantly longer in the RA group (mean 10, range, 6–16 weeks) compared with the prosthesis group (mean 6, range, 2–12 weeks) (95 % CI: 1.9 to 5.1; p = 0.0001).

### 3.4 Postoperative thumb length restoration

**Fig. 5:**
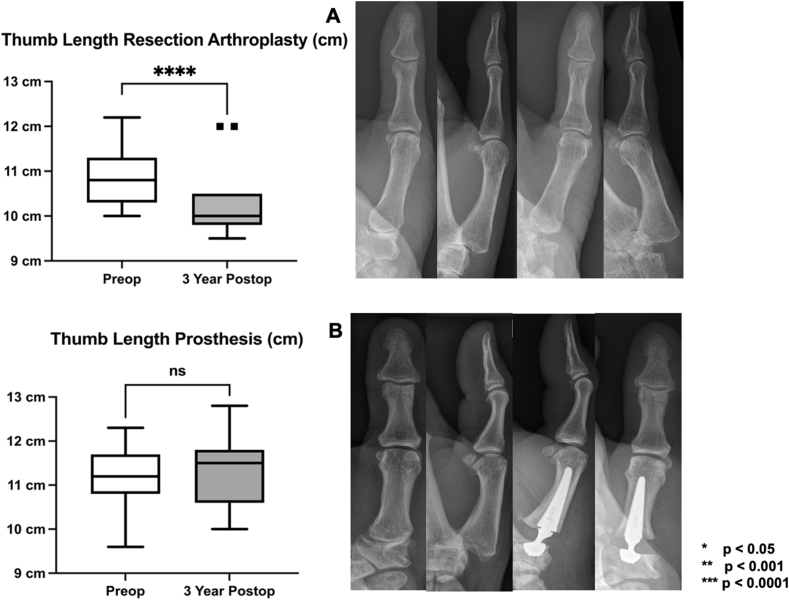
Boxplot graphs (minimum, 25th percentile, median, 75th percentile, maximum) illustrate the comparison of preoperative and postoperative comparison of thumb length restoration for (A) resection arthroplasty and (B) dual mobility prosthesis 3 years after surgery.

Radiographical thumb length measurements demonstrated that thumb length was significantly shortened due to the operative procedure in the RA group (preoperative: 10.9, range: 10.0–12.2 cm vs 3 year postoperative: 10.3, range 9.5–12.0 cm; 95 % CI: 0.4 to 0.9; p < 0.0001). In comparison, thumb length in the prosthesis group remained consistent throughout the follow-up examinations (preoperative: 11.1, range: 9.6–12.3 cm vs 3 year postoperative: 11.32, range 10.0–12.8 cm; 95 % CI: 0.1 to 4.1; p = 0.2) (Fig. 5).

### 3.5 Postoperative complications

**Fig. 6:**
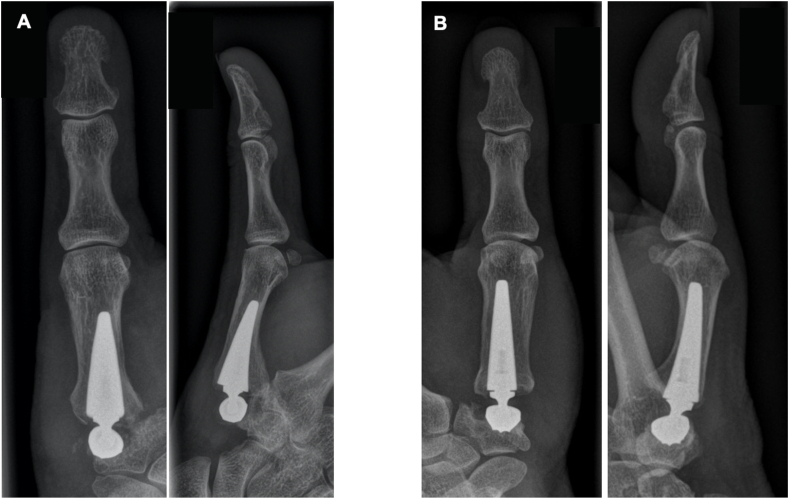
(A) Postoperative radiograph of a 56-year-old man presenting prosthesis dislocation with breakout of the trapezium. (B) Presentation of a bony cyst around the cup at 3-year follow-up after prosthesis implantation.

During follow-up examination, in one patient prosthesis dislocation (2 %) occurred after 31 months. A conversion to Lundborg's RA was necessary. No operative revisions were required due to infection, loosening, or dislocation in the prosthesis group, nor did we see a severe proximalisation of the first metacarpal in the RA group. Radiographs did reveal a small bony cyst formation around the cup in two thumbs (4 %). However, clinically prosthetic stability was not affected (Fig. 6).

## 4 Discussion

Surgery for TMC joint osteoarthritis is effective to relieve pain and improve manual function. However, there is currently no consensus regarding the most effective surgical technique.^11^ Resection arthroplasty is regarded the surgical standard procedure.^12^ In some cases RA can cause functional impairment, thumb shortening, or even proximalisation of the metacarpal onto the scaphoid leading to painful scaphometacarpal arthritis.^13^, ^14^, ^15^ Dual mobility prosthesis may represent a promising less ablative, joint function preserving alternative. With respect to current literature, this study is the first to prospectively compare mid-term follow-up outcomes of RA with a dual mobility prosthesis. Our major finding is that postoperative pinch strength was significantly stronger in the prosthesis group at all timepoints of examination. This might be related to the preserved length and the thumb's biomechanical stability.

Our results indicate a significantly longer period from surgery to achieving complete pain relief in the RA group. This might be due to the invasiveness of the RA procedure. In addition, a significantly longer interval from surgery to achieving comparable results for radial and palmar abduction was observed in the RA group in comparison to the prosthesis cohort. An explanation could be the longer postoperative immobilization period in the RA group, which is necessary to let the resection space be filled up by fibrotic tissue and scar tissue providing the required stability of the former joint area. As a result, the duration of disability was significantly longer in the RA group, posing a particular challenge for younger patients. The longer period of disability notably extends the time returning to work in the RA group.

Our surgical results of RA for functional recovery and pinch strength were comparable with those reported in the literature.^16^ However, studies focusing on pinch strength after dual mobility prosthesis implantation revealed superior outcomes compared to those reported for RA.^5^^,^^6^ Some authors suggest that the shortening of the thumb after RA may result in reduced pinch strength.^14^ This was illustrated by our results. We were able to demonstrate that prosthesis implantation could preserve the original thumb length. Therefore, we conclude that thumb length preservation positively correlates to the superior pinch strength in the prosthesis group. We assume that the significant better results of the functional scores QuickDASH and MHQ may be due to the maintained stability of the thumb.

In our study the one single complication requiring surgical revision occurred in the prosthesis group. This patient underwent revision surgery because of late prosthesis luxation and was converted to Lundborg's resection arthroplasty. Anyhow, this case demonstrates that secondary conversion to conventional RA is still possible after prosthesis implantation. It is long known that secondary RA after failed TMC joint arthroplasty often leads to satisfactory results as compared to primary RA.^17^^,^^18^ In another prospective study Lussiez et al. examined the outcome of the dual mobility TOUCH® prosthesis, and reported the appearance of de Quervain's tenosynovitis (4x) and of trigger thumb (6x) between 3 and 12 months postoperatively.^6^ However, in our cohort we were never confronted with either disease. Maybe this is due to our surgical approach in both procedures in which the extensor pollicis brevis tendon is always released on its whole length over the anatomical snuff box.

Between 6 and 12 months after prosthesis implantation, we observed the presence of a small bony cyst in the trapezium in two patients. Neither of the patients reported any clinical complaints. Therefore, this was not classified as a complication since the cups remained fully anchored within the trapezial cavity. This may either occur due to periprosthetic remodeling of non-cemented prostheses or to an uneven preparation of the trapezial cavity.^6^^,^^19^^,^^20^

Although our study yields promising results, it is important to consider several limitations. First, our study did not reach a mean of 5 years follow-up as it is recommended for an established implant.^21^ More interesting will be the results after 5 years and 10 years or even more. Second, the study groups could not be randomized because from the early days of dual head TMC joint protheses too many patients presented asking for implantation. This also caused the different sizes of the groups, and both might influence statistical outcome parameters. In addition, we assume that implantation of the prosthesis may also be feasible with a trapezium height of less than 9 mm.

In conclusion, both surgical techniques for painful TMC joint osteoarthritis show high patient satisfaction at mid-term follow-up. Implantation of a dual-mobility TMC joint prosthesis appears to be associated with superior pinch strength and shorter recovery time, as well as better restoration of ROM and earlier return to work compared to patients after trapezium RA.

## Funding

KeriMedical® paid for the Ethics Committee fee and reimbursed the patient's travel expenses.

## Ethical approval

Was granted by the (Ethics Committee Mainz, Rheinland-Pfalz (2019–14184).

## Informed written consent

Was obtained from all patients enrolled in the study.

## CRediT authorship contribution statement

**Florian Falkner:** designed the study, performed statistical analyses, data interpretation, and wrote the, Writing – original draft, of the manuscript. **Arman Mahmut Tümkaya:** reviewed the patients, performed data acquisition and curation, and assisted with data interpretation. **Benjamin Thomas:** Supervision, statistical analyses, assisted with data interpretation, language editing, and manuscript revision. **Arne Böcker:** helped in data interpretation, manuscript revision and, Writing – review & editing. **Martin Aman:** helped in data interpretation, manuscript revision and, Writing – review & editing. **Berthold Bickert:** performed the surgery as a senior surgeon, supported data acquisition and manuscript revision. **Leila Harhaus:** designed and, Supervision, the study, and performed the surgery as a senior surgeon. **Benjamin Panzram:** additionally, Supervision, the study at the University of Heidelberg, helped with, Formal analysis, and interpretation and wrote the final version of the manuscript.

## Declaration of competing interest

Two authors declare a conflict of interest.

BP received payment for a scientific lecture and a surgical workshop regarding the Touch prosthesis by Medartis. LH received a research grand independandly from this study by KeriMedical. All other authors declare no potential conflicts of interest with respect to the research, authorship, and/or publication of this article. During the preparation of this work none of the authors used generative AI or AI-assisted technology in the writing process.
