# Supplementary material for: Resection arthroplasty versus dual mobility prosthesis in the treatment of trapeziometacarpal joint osteoarthritis: A 3 year non-randomized prospective study
Source: J Orthop. 2024 Jun 10;57:72–8. doi: 10.1016/j.jor.2024.06.005 (PMC11231515; doi:10.1016/j.jor.2024.06.005)
Supplement: Multimedia component 1 [file mmc1.docx]

**Table S1.** Summary of values for pain, ROM, and strength between resection arthroplasty (RA) and prosthesis (P) during follow-up examinations.

| Assessment | Follow-up in months | | | | | | | | | | | | | |  |
| --- | --- | --- | --- | --- | --- | --- | --- | --- | --- | --- | --- | --- | --- | --- | --- |
|  | Preop | | 1.5 | | 3 | | 6 | | 12 | | 24 | | 36 | |  |
|  | RA | P | RA | P | RA | P | RA | P | RA | P | RA | P | RA | P |  |
| VAS for pain | 8 (1) | 7 (1) | 4 (2) | 2 (2) | 3(2) | 1(1) | 1 (1) | 1 (1) | 1 (1) | 1 (1) | 1 (1) | 1 (1) | 1 (1) | 1 (1) |  |
| MCP ex/flex | 45 (8) | 46 (5) | 40 (8) | 45 (4) | 42 (9) | 47 (5) | 44 (9) | 49 (5) | 45 (9) | 49 (6) | 45 (9) | 49 (6) | 45 (9) | 49 (6) |  |
| TMC rad. abd | 36 (4) | 38 (3) | 28 (7) | 36 (4) | 33 (7) | 42 (4) | 40 (6) | 45 (4) | 42 (4) | 46 (4) | 42 (5) | 47 (4) | 43 (4) | 47 (4) |  |
| TMC pal. Abd | 32 (3) | 39 (5) | 28 (7) | 36 (5) | 33 (7) | 48 (5) | 37 (4) | 48 (5) | 43 (4) | 48 (5) | 44 (4) | 48 (5) | 45 (4) | 48 (5) |  |
| Kapandji ind. | 7 (2) | 7 (1) | 8 (1) | 8 (1) | 9 (1) | 9 (1) | 9 (1) | 9 (1) | 9 (1) | 9 (1) | 10 (1) | 10 (1) | 9 (1) | 9 (1) |  |
| Grip strength | 15 (9) | 13 (7) | 11 (8) | 12 (7) | 12 (9) | 17 (8) | 13 (9) | 18 (8) | 14 (8) | 19 (9) | 14 (8) | 20 (9) | 14 (7) | 20 (9) |  |
| Pinch strength | 4 (2) | 4 (1) | 2 (2) | 4 (2) | 3 (2) | 5 (2) | 3 (2) | 5 (2) | 3 (2) | 6 (2) | 3 (1) | 6 (2) | 3 (2) | 6.0 (2) |  |

Data presented as mean (standard deviation). MCP: metacarpophalangeal joint; P: prosthesis; RA: resection arthroplasty; TMC: trapeziometacarpal joint; VAS: visual analogue scale.
